# New Sensors for Monitoring pH and Corrosion of Embedded Steel in Mortars during Sulfuric Acid Attack

**DOI:** 10.3390/s22145356

**Published:** 2022-07-18

**Authors:** Rui Sampaio, Alexandre Bastos, Mário Ferreira

**Affiliations:** CICECO—Aveiro Institute of Materials, DEMaC—Department of Materials and Ceramic Engineering, University of Aveiro, 3810-193 Aveiro, Portugal; ruisampaio@ua.pt (R.S.); mgferreira@ua.pt (M.F.)

**Keywords:** sulfuric acid attack, mortar, pH sensors, corrosion monitoring, electrochemical impedance spectroscopy

## Abstract

The sulfuric acid attack is a common form of degradation of reinforced concrete in contact with industrial wastewater, mine water, acid rain, or in sewage treatment stations. In this work, new pH-sensitive IrOx electrodes were developed for monitoring the pH inside mortar or concrete. To test their ability, the pH sensors were embedded in mortar samples at different depths and the samples were exposed to sulfuric acid solution. In another set of experiments, iron wires were placed at the same depths inside similar mortar samples and their corrosion was monitored as the acid attacked the mortar. Severe acid attack led to cement dissolution and formation of gypsum. The new pH sensors succeeded in measuring the pH changes inside the mortars. The pH gradient, from the high acid environment to the high alkaline mortar interior, occurred in a narrow region. Corrosion of the iron electrodes started only when the acidic solution was in their close vicinity.

## 1. Introduction

Cement-based materials are the most used construction materials in the world. They are stable in a wide range of natural and service environments, but their durability decreases when reinforced with steel, particularly in some particularly aggressive environments [1,2]. One such case is sulfuric acid attack [2,3,4,5,6,7,8,9,10,11,12,13,14], which can be found in industrial wastewater, sewage treatment stations and pipe systems, mines, and acid rain. It combines acid attack and sulfate attack [2,3]. In sulfuric acid attack the main components of Portland cement hydrates—portlandite and calcium silicate hydrates (CSH)—are dissolved by the acid and react with sulfate, forming calcium sulfate dihydrate (gypsum):

H_2_SO_4_ + Ca(OH)_2_ → CaSO_4_·2H_2_O (1)

The calcium sulfate reacts with the aluminates present in the cement matrix leading to the formation of ettringite:

3CaSO_4_ + 3CaO·Al_2_O_3_·6H_2_O + 26H_2_O → Ca_6_Al_2_(SO_4_)_3_(OH)_12_·26H_2_O (2)

Volume expansion of the reaction products leads to the cracking of concrete. The rate of acid attack is influenced by many factors, including environmental factors (type of acid, concentration, surface abrasion, fluid dynamics), material factors (types of cement, aggregates, admixtures), and fabrication factors (water/cement ratio, curing time, compactness). The deterioration is faster in lower pH [6,7]. The surface abrasion increases the degradation rate due to the removal of reaction products, which, when accumulated on the surface, could form a protective layer limiting the acid diffusion [8]. The thickness of this layer depends on the dynamics of the contacting fluid [9]. Factors related to the fabrication of concrete mainly affect porosity and compressive strength [10]. Porosity allows the diffusion of chemical species through the concrete matrix and increases the contact area between acid and concrete, accelerating the process. On the other hand, higher porosity decreases the appearance of cracks because of the easier accommodation of the expansion caused by the formation of gypsum [11,12]. In general, supplementary materials like fly ash improve the resistance to acid attack by decreasing the porosity of the cementitious matrix [7,13]. The aggregates may or may not resist to acid attack. Quartz, which is the main constituent of sand and is present in many aggregates, withstands acid attack and therefore increases the tortuosity of the diffusion path of ions through the attacked layer. Conversely, it lacks neutralization capacity [6]. 

The evaluation of sulfuric acid attack is commonly achieved by weight loss, thickness reduction measurement, quantification of reacting species (calcium, sulfate, and pH), and determination of the compressive strength under several experimental conditions [2], including constant pH and constant sulfate methods [7]. The main methods used for determining the pH of the pore solution of mortar and concrete are the pore solution extraction method, the in-situ leaching method, and the ex-situ extraction method [15]. In the pore solution extraction, a high pressure is applied to the material to squeeze out the solution for measurement [16,17]. In the in-situ leaching method, a hole is drilled in the concrete, the hole is filled with distilled water, and the pH is measured after sufficient time of equilibration [18,19]. The ex-situ extraction consists of grinding the mortar or concrete to fine powder, mixing it with decarbonated distilled water, and measuring the pH after an equilibration time [20,21,22,23]. pH indicators are also used particularly for determining carbonation in concrete [24,25]. Embedded sensors allow nondestructive measurement of the pH inside concrete. They have the possibility of following the pH variation in real time. A few sensors have been used to determine the pH in cementitious samples, including fiber optic and electrochemical sensors [26,27,28,29,30,31]. These electrochemical sensors are metal|metal oxide electrodes (mostly iridium, but also silver, titanium, ruthenium, and others). The sensing principle is based on their potentiometric response, where a reversible redox reaction occurs between the metal oxide and H^+^. A linear response exists between the potential of the electrode and the pH. These electrodes are robust, with fast response, and permit fabrication in a range of shapes and sizes, from microsensors to macrosensors.

The determination of the pH variation of the pore solution during sulfuric acid attack is not documented in the literature. Moreover, the studies concerned with sulfuric acid attack generally focused on the effect on the concrete, while the steel reinforcement is seldom considered. Just one work was found where the open circuit potential of the reinforcing steel was monitored, but the acid front did not reach the steel [11]. 

The present work contributes to the research of the sulfuric acid attack on reinforced concrete by studying the pH change and corrosion of iron wires inside mortar in contact with a high concentrated sulfuric acid solution. The pH was monitored by iridium oxide electrodes specially developed for this work. This type of potentiometric sensors based on metal oxides are well suited because they are mechanically robust and chemically stable with easy miniaturization and inexpensive production. The pH sensors and the Fe wires were embedded in mortar samples at different distances from the exposed surface and their open circuit potential (OCP) monitored during the sulfuric acid attack. The corrosion of the Fe wires was also investigated using electrochemical impedance spectroscopy (EIS).

## 2. Materials and Methods

### 2.1. Synthesis and Characterization of pH IrOx Sensors

pH-sensing potentiometric sensors were made by electrodepositing an IrOx film onto 316L stainless steel wire with 0.8 mm diameter (Goodfellow, Huntingdon, UK). Each wire was abraded down to SiC grit 4000 and ultrasonically cleaned in acetone for 5 min. Then, it was connected to an electrically conductive wire through colloidal silver suspension (PELCO 16034, Ted Pella, Redding, CA, USA)). The electrical connection between the wires was isolated and reinforced with epoxy resin and Lacomit varnish (AGG371, Agar Scientific, Stansted, UK). Finally, the synthesis of the pH sensitive films was performed using cyclic voltammetry, sweeping the potential between −0.3 and +0.8 V vs. Red Rod electrode (199 mV vs. SHE at 25 °C, Radiometer Analytical, Lyon, France) at a scan rate of 50 mV/s for 50 cycles in a growth solution prepared according to the Yamanaka method [32]. First, 0.15 g of IrCl_4_ were dissolved in 100 mL of deionized water and mixed with magnetic stirrer for 30 min. Then, 0.5 g of oxalic acid was added as complexing agent to prevent precipitation of IrO_2_ in alkaline medium [33], followed by 10 min of stirring before adding 1 mL of H_2_O_2_ (30%). After 10 more minutes of stirring, the pH was raised to 10.5 by the slow addition of sodium carbonate [33,34]. This addition prevents the passivation of the stainless steel wire. The solution was stored for a few days, avoiding contact with light, and it was used while it had a blueish color. The film growth was performed with an Autolab PGSTAT 302N (Methrom Autolab, Utrecht, The Netherlands) potentiostat, with the stainless steel wires as working electrodes, a platinum wire as counter electrode, and the Red Rod electrode as reference. The electrochemical cell was inside a Faraday cage, and the solution was at room temperature, quiescent, and open to air. 

The potentiometric response of each single sensor was determined with commercial pH buffer solutions (Fluka) in the pH range from 2 to 13. The open circuit potential (OCP) of each sensor was continuously measured while the pH varied stepwise with the buffer solutions. The resulting calibration curve (potential vs. pH) allowed relating potential measurements with the pH of the environment in contact with the sensor. The reference electrode (RE) used in these experiments was a saturated calomel electrode (SCE).

### 2.2. Mortars and Sensors Embedment

**Figure 1:**
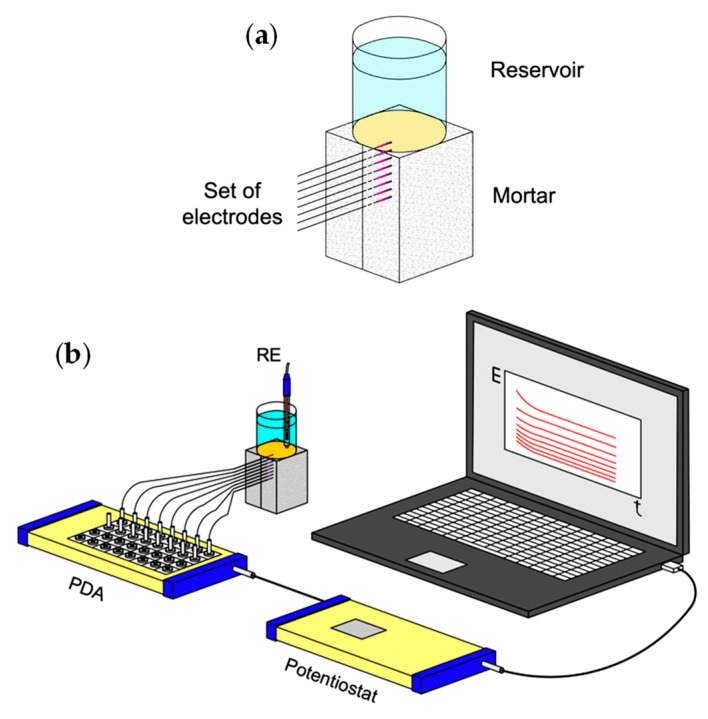
(**a**) Sketch of the pH sensors or Fe wires embedded in the mortar sample. (**b**) Experimental set-up for monitoring the pH or the corrosion potential of Fe wires inside mortar during exposure to the sulfuric acid solution.

Mortar samples of 6 × 5 × 5 cm^3^ were prepared with ordinary Portland cement (CEM I 42.5 N), sand, and water. The composition is presented in Table 1. The amount of cement was lower than the usual to induce higher porosity and faster degradation. A set of eight pH sensors with a separating distance of 5 mm was assembled in the mortar samples—Figure 1a. After casting, the samples were cured for 1 week in the molds and 2 weeks in distilled water after demolding. A polypropylene tube was glued to the top of each sample, to be filled with the testing solution. The remaining faces were isolated with an epoxy coating. 

### 2.3. pH Monitoring

The sulfuric acid attack was simulated by filling the polypropylene tube with 1 M H_2_SO_4_, a quite high concentrated solution, chosen with the objective of obtaining fast degradation. The pH monitoring started immediately. The solution was daily renewed, and the loose debris resulting from the destruction of the mortar removed with the help of a plastic pipette. The pH evolution within the mortar samples was determined potentiometrically, by measuring the OCP of the sensors against a saturated calomel electrode. The potentials were converted to pH using the calibration curve of each single sensor. Since the sensors were to operate inside mortar, the calibration was made after the sensors stayed immersed in saturated Ca(OH)_2_ solution (pH ≈ 12.6) during 15 days. The calibration followed the procedure described in Section 2.1. All sensors presented similar calibration curves.

The measuring setup is presented in Figure 1b. It was constituted by a CompactStat potentiostat (Ivium Technologies, Eindhoven, The Netherlands) coupled to a Ivium peripheral differential amplifier (PDA) with eight channels (10^12^ Ω input impedance) for simultaneous measurements. The products from the sulfuric acid attack were analyzed by X-ray diffraction (XRD) using a diffractometer (PANalytical XPert-Pro, Almelo, The Netherlands) with CuKα radiation (λ = 1.54060 nm), operating at a scan rate of 0.01º/s.

### 2.4. Corrosion Monitoring

Mortar samples like the ones described above and depicted in Figure 1a were produced with 1 mm diameter iron wires (99.5% pure, Goodfellow, Huntingdon, UK) in the place of the IrOx pH sensors. 1 M H_2_SO_4_ filled the solution reservoir and the arrangement sketched in Figure 1b was used to monitor the corrosion potential of the iron wires. At given times, EIS measurements were made on individual Fe wires using a Gamry Reference 600 equipment, with a 10 mV rms potential perturbation around OCP, in the frequency range from 100 kHz to 1 mHz, with 7 points per decade with logarithmic distribution. The Fe wires were the working electrodes, a saturated calomel electrode was the reference, and a platinum wire was the counter electrode.

## 3. Results and Discussion

### 3.1. Characterization of the pH Sensors

**Figure 2:**
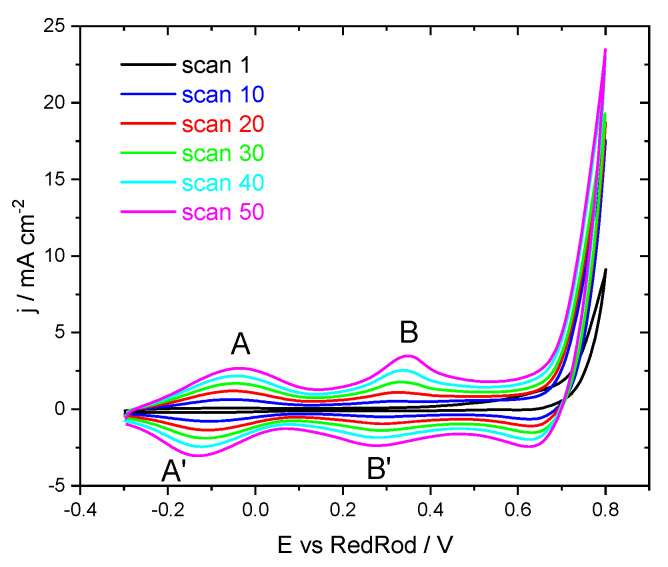
Cyclic voltammograms of IrOx growth with a scan rate of 50 mV/s.

Figure 2 presents cyclic voltammograms of the growth of the pH-sensitive IrOx films. The voltammograms presented well-defined redox peaks—A/A′ and B/B′—which are commonly assigned to the redox couples Ir(III)/Ir(IV) and Ir(IV)/Ir(V), respectively [34]. The currents increased with the scan number reflecting the film growth. 

**Figure 3:**
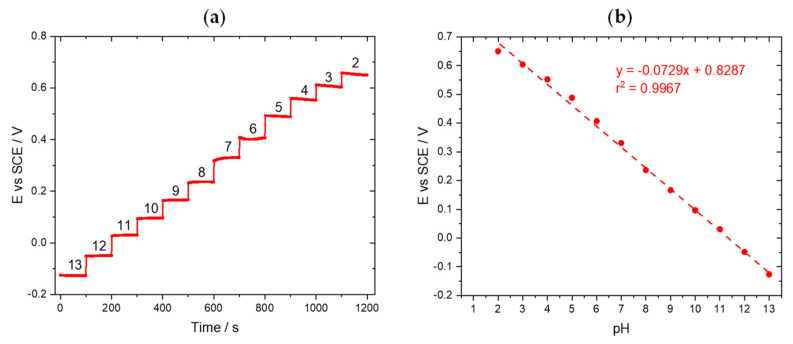
(**a**) Potentiometric response to pH and (**b**) corresponding calibration curve of an IrOx sensor.

The potentiometric response of the pH sensors (316L stainless steel wires coated with the IrOx film) and respective calibration curve are represented in Figure 3. The sensors showed a fast and stable response to the pH variation, with a super-Nernstian slope of 72.9 mV/pH, attributed to the hydration state of iridium oxides, a characteristic of electrochemically synthesized IrOx films [35]. The electrochemical equilibrium is attributed to the following chemical reaction [28,36].

2[IrO_2_(OH)_2__−_
*_x_*·(2 + *x*)H_2_O]^(2^^−^
*^x^*^)^^−^ + (3 − 2*x*)H^+^ + 2e^−^ <=> [Ir_2_O_3_(OH)_3_·3H_2_O]^3^^−^ + 3H_2_O (3)

where *x* varies from 0 to 0.5. In this reaction, more than one H^+^ is involved in the exchange of each electron, which results in the super-Nernstian slope. Modifications in the hydration state of the surface, will change the value of *x* and, consequently, the slope of the electrode potential response.

### 3.2. Measurement of pH Inside Mortar Samples

**Figure 4:**
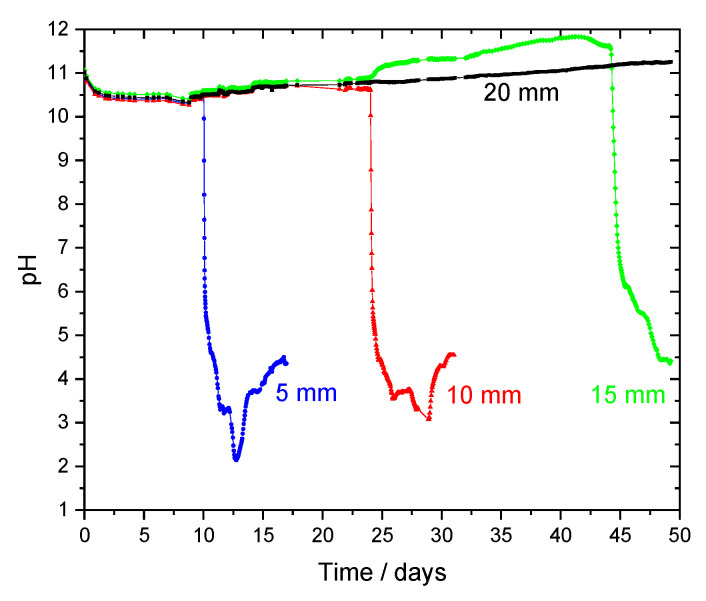
Mortar pH measured by the sensors at different depths: 5, 10, 15, and 20 mm.

Figure 4 shows the pH measured by the embedded sensors. The use of an array of sensors placed at different depths allowed monitoring the evolution of the pH inside the mortar in contact with the sulfuric acid. Initially all sensors presented similar, stable, and high pH readings. The first change was detected after 10 days of testing, by the sensor at a depth of 5 mm showing a significant drop in pH. During this time, the surface exposed to the acid solution changed from the typical grey to white. In addition, the surface started revealing debris, mostly sand and products of the neutralization reactions of the binder and the sulfuric acid. The accumulated loose debris was removed with a plastic pipette. The first sensor became visible one day after the beginning of the pH drop. This indicates that the transition zone between the acid front and the intact mortar is narrow. Similar pH decrease (fast once started) was detected by the second sensor (10 mm depth) after 24 days. The third sensor (15 mm depth) registered the pH drop after 44 days. At this stage, the response of the sensors located at greater depths (20, 25, 30, 35, and 40 mm) remained constant and in the alkaline region, showing that the pH inside the mortar was not affected by the high external acidity. The mortar reacted with the acid solution and was dissolved, but only a narrow region at the mortar boundary was affected. There is no pH gradient in the mortar except for just the first 1 to 2 mm from the interface with the solution. The reason is that, despite the strong acid solution, the alkaline composition of the mortar reacted with the acid, neutralizing it at the expense of its partial dissolution. Mainly sand resisted dissolution, and a white deposit of gypsum was formed—Equation (1). Part of the white layer was removed, but its thickness grew with time, which explains the longer time for the response of the third sensor. The layer provided some barrier to the solution and acid progression. After the initial fast decrease, the pH reached values between 3–4, when it stabilized for about one day. Then, it decreased again, followed by an increase in the following days. This increase is an artifact, discussed in the next section. 

**Figure 5:**
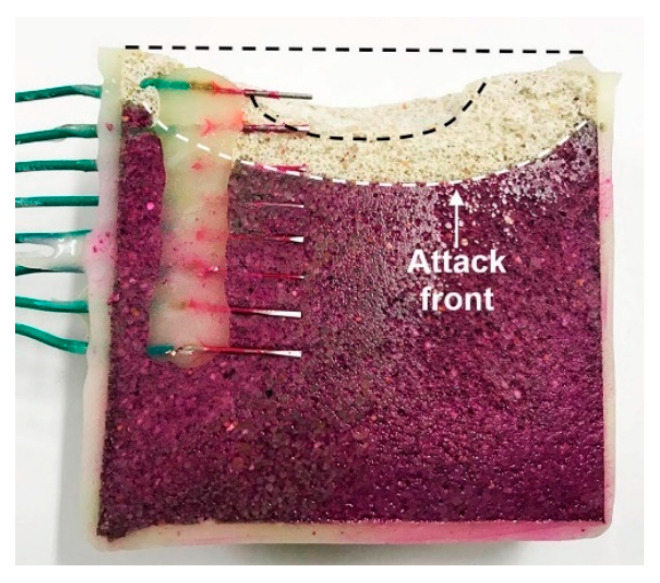
Cross section of mortar sample after 50 days of acid attack. Red color is due to the presence of phenolphthalein identifying the region with pH > 9.

The test was stopped after 50 days, and the sample was cut parallel to the sensors (Figure 5). Phenolphthalein solution helped visualize the internal pH of the sample [24,25]. The pH of the bulk of the mortar remained high despite the acid attack, except for a narrow region at the surface, which is in good agreement with the data provided by the sensors.

Afterward, parts of the remaining mortar and the gypsum layer were ground for measuring their pH by the ex-situ extraction method [15,20,21]. For that purpose, the same amount of powder and of decarbonated distilled water (boiled and bubbled with argon to remove carbon dioxide) were mixed by magnetic stirring in a closed container during 24 h. The pH was measured by potentiometry with an Inlab Expert Pro pH combined electrode and a SevenMulti meter, both from Mettler Toledo (Columbus, OH, USA). The values obtained were 12.3 ± 0.2 for the mortar and 7.9 ± 0.1 for the gypsum layer. The pH of the mortar is higher than the measured by the IrOx sensors, but it is expected that the pH increases with time as a results of cement hydration [1]. Such pH increase is observed in the results of the sensors placed at 15 and 20 mm shown in Figure 4.

**Figure 6:**
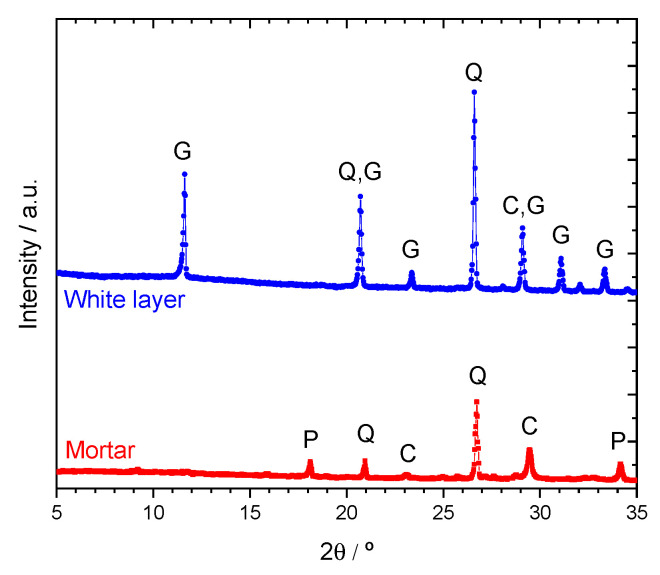
Diffractograms of mortar before testing (Mortar) and the white layer at the top surface of the sample (White layer). P—portlandite, Q—quartz, C—calcite, and G—gypsum.

The white layer was analyzed by XRD and compared with the mortar before testing. The diffractograms are presented in Figure 6. The main components of mortar—calcite, portlandite, and quartz—were detected in the XRD analysis of the sample before attack. The white layer showed the presence of gypsum and quartz (sand from the mortar). The phase assignment was validated with International Centre for Diffraction Data (ICDD) cards for quartz (01-085-0798), portlandite (01-076-0571), calcite (04-023-8700), and gypsum (04-008-9805), using the HighScore Plus software from Malvern Panalytical.

### 3.3. Response of IrOx Sensors in 1 M H_2_SO_4_

**Figure 7:**
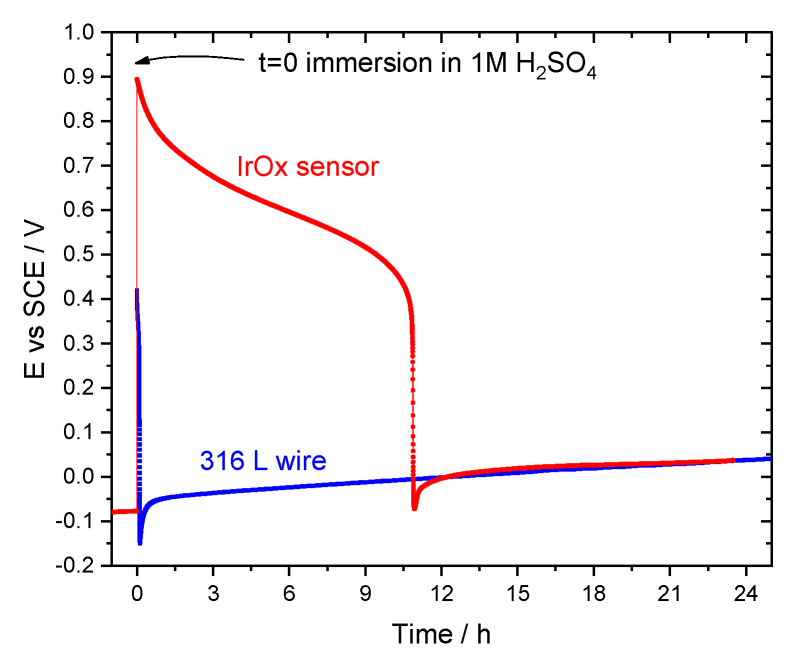
OCP evolution of IrOx sensor initially immersed in Ca(OH)_2_ saturated solution (t < 0) and then changed to 1 M H_2_SO_4_ (t > 0), compared to substrate (316L wire) response in 1 M H_2_SO_4_.

The response of the IrOx pH sensor during the sulfuric acid attack was simulated by placing it for a few days in saturated Ca(OH)_2_ solution (pH ≈ 12.6) to mimic the mortar pore solution and then moving to 1 M H_2_SO_4_ solution while measuring the potential. The result is presented in Figure 7. The potential was stable in the alkaline environment (around −0.080 V vs. SCE, corresponding to a pH = 12.45 using the calibration curve in Figure 3b. As soon as the electrode was placed in the acid solution, the potential immediately changed to a high positive value, consistent with the response in acid conditions. Then, it decreased steadily for a few hours until an abrupt drop to values typical of the corrosion potential of bare 316L substrate. This was confirmed by measuring the potential of the 316L wire in the same solution, also shown in Figure 7. The strong acidic environment promoted a gradual dissolution of the IrOx film with a simultaneous change in the measured potential.

These results can explain the “increase” in pH verified in sensors at 5 and 10 mm after the initial pH drop (Figure 4). Due to the dissolution of the mortar, the sensors became exposed to strong acidic conditions and the IrOx layer was dissolved. As a result, the measured potential was that of the stainless steel substrate and no longer correlated with the pH. The application of the calibration curve to these new potential values gave an apparent but erroneous increase of pH. There is no IrOx film; consequently, the calibration curve is no longer applicable.

### 3.4. Corrosion of Iron Wires in Mortar during Sulfuric Acid Attack

**Figure 8:**
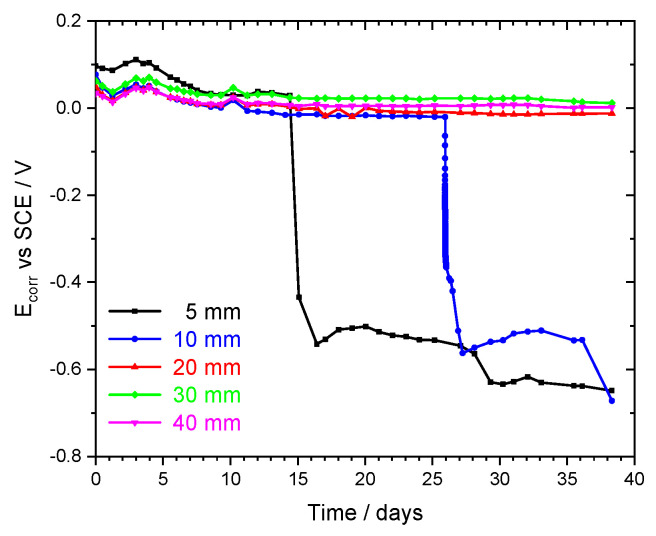
OCP evolution of Fe electrodes embedded in a mortar sample exposed to 1 M H_2_SO_4_.

Up to this point, this paper focused on the mortar degradation by the sulfuric acid. Now, the attention will be drawn to the effect on iron wires embedded in the mortar, which simulate steel bars in reinforced concrete. The evolution of the corrosion potential of the iron wires at different depths in mortar is presented in Figure 8. Initially, the iron wires were protected by a passive layer and showed constant and high potential values, in the range of 0 to 0.1 V vs. SCE. The acid solution did not affect them until it was close to their surface. When it occurred, the drop in potential was sudden and fast, reaching −0.360 V vs. SCE in the first 2 h. Then, the potential decrease became slower, taking about 36 h to reach −0.55 V vs. SCE. A two-step evolution was also observed in the pH measured with the IrOx sensors. The initial drop occurred when the electrodes were covered by a layer of mortar/gypsum, and the second drop occurred with the disappearance of that layer. The changes in potential took place after 14 days and 26 days for the wires at 5 mm and 10 mm, respectively. These times were slightly longer than those found for the pH decrease in Figure 4, which is explained by the variability inherent to this type of samples and also by a possible slight difference in depths of the sensors in each sample.

**Figure 9:**
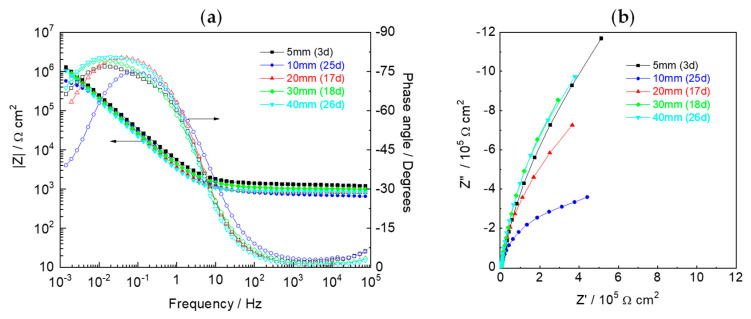
EIS response—(**a**) Bode plots and (**b**) Nyquist diagrams—of Fe wires embedded in the mortar before being reached by the acid front.

Electrochemical impedance spectroscopy was used to monitor the state of the Fe wires at different moments of the acid attack. Figure 9 presents the impedance of electrodes at depths of 5, 10, 20, 30, and 40 mm, before being reached by the acid front. The response was similar, with essentially two regions, a resistive one at high frequencies, and a capacitive one at middle and lower frequencies.

The response at higher frequencies is due to the resistance of the H_2_SO_4_ solution (small because of its high conductivity) in series with the resistance of the pore solution network in the mortar. The response at middle and lower frequencies comes from the capacitance of the passive film or the double layer capacitance for longer times. For the sake of comparison and to avoid considering different equivalent electric circuits, it was decided to use a simple generic circuit represented by R_HF_(CPE_LF_R_LF_), to numerical fit the experimental data, where R_HF_ represents the resistance at higher frequencies, CPE_LF_ is a constant phase element used to express the capacitive response, and R_LF_ is the resistance at lower frequencies. It can be either the passive film resistance or the charge transfer resistance, depending on the evolution stage. The fitting was performed with the ZView program (Scribner Associates, Southern Pines, NC, USA) and the results are presented in Table 2. The values were similar for all wires because they correspond to the same material in the same environment. The variations at the lower frequencies are considered to be the manifestation of the variability in the passivity condition of the Fe wires. The impedance measurement is sensitive to small defects that may exist in a high impedance barrier. Hence, the difference in impedance at the low frequencies can indicate small differences in the passivity of the wires, due to, for example, small surface heterogeneities in composition or morphology that do not allow a full passivation in those points.

**Figure 10:**
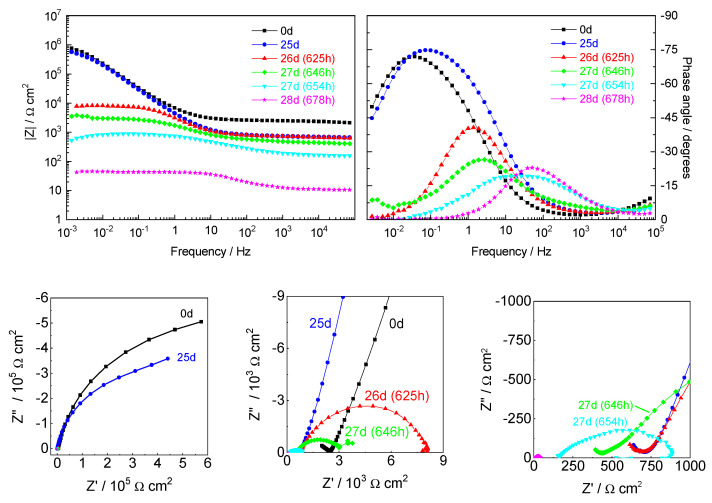
EIS response (Bode diagrams and Nyquist plots) of the Fe wire at 10 mm in different stages of the acid attack.

The impedance response changed when the acid front reached the Fe wires. This is shown in Figure 10 for the wire located 10 mm inside the mortar. The impedance was similar during the first 25 days. The only variation was the decrease of the resistance at high frequencies, due to the reduction of the mortar thickness, as a result of its gradual dissolution by the acid. The remaining part of the spectra, the part related to the Fe response, showed no changes. Then, between days 26 to 28, a fast decrease in impedance was observed, easily noticed by the strong decline of resistances R_HF_ and R_LF_ (Table 3). The first, at higher frequencies, was related to the mortar cover, which disappeared and left the wire directly exposed to the acid solution. The decrease of R_HF_ revealed the fast dissolution of the mortar cover until the wire became totally exposed in day 28. The resistance at lower frequencies was associated with the response of the passive film at the beginning (R_pass_), followed by the loss of passivity by the action of the acid and the active corrosion afterward (R_ct_). Therefore, R_LF_ was a measure of the corrosion resistance of the iron wire. The corrosion rate was so intense in this last stage that the Fe wire has dissolved in just one day.

**Figure 11:**
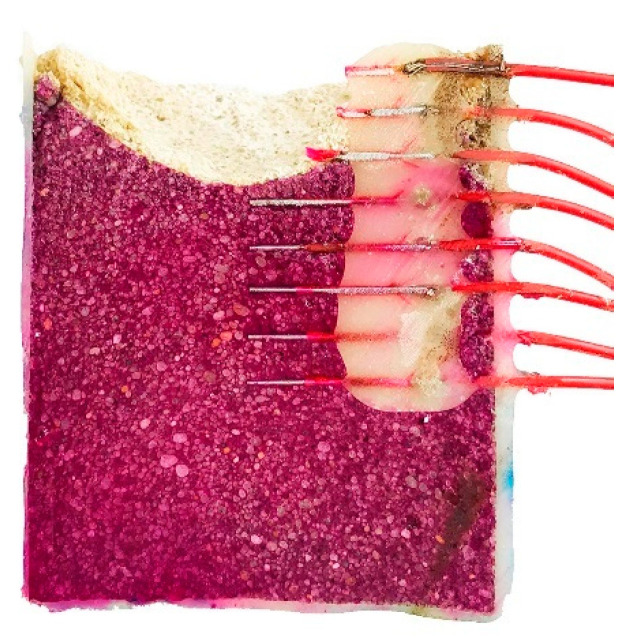
Cross-section of mortar sample with Fe wires after 39 days of acid attack. Red color is due to the presence of phenolphthalein identifying the region with pH > 9.

At the end of the experiments, the mortar was cut (Figure 11). It resembles Figure 5, with the red color of phenolphthalein identifying the alkaline environment coincident with the mortar that remains intact. The pH shift was abrupt, from the high alkaline (red) to the high acidic regions (white), without transition region. The steel wires were passive inside the mortar and active, with total dissolution, when exposed to the acidic environment. 

**Figure 12:**
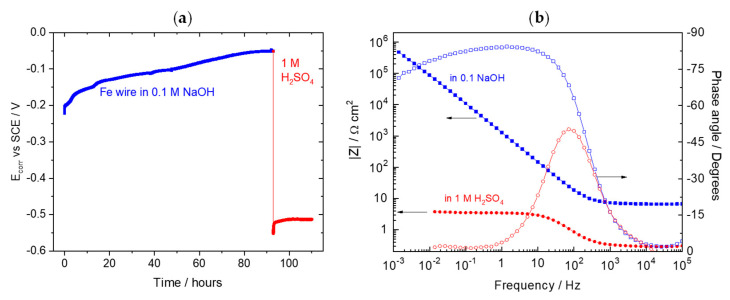
(**a**) OCP evolution of iron with solution change and (**b**) EIS response of an iron wire immersed in 0.1 M NaOH (blue) and then immersed in 1 M H_2_SO_4_ (red).

To compare the response of the Fe wire in the alkaline and acid environments, one Fe wire was placed in 0.1 M NaOH for a few days. The open circuit potential (Figure 12a) increased continually (the passive film was growing, even after 4 days of exposure to the high alkaline solution) and reached −0.05 V vs. SCE when the alkaline solution was replaced by the sulfuric acid. The potential immediately decreased to −0.55 V vs. SCE, the value found in the Fe wires during the acid attack. Impedance measured in the two environments (Figure 12b) was in line with the spectra presented in Figure 10. The impedance in alkaline environment resembled the spectra measured in the first days of testing, just with a much smaller R_HF_, coincident with the solution resistance in absence of the mortar cover. The impedance in 1 M H_2_SO_4_ was like the one measured at day 28 (same electrode and same environment).

## 4. Conclusions

This work investigated the degradation of mortar samples with embedded pH sensors and Fe wires (to simulate bars of reinforced concrete) exposed to a strong sulfuric acid solution, which emulates conditions found in some harsh environments. The pH sensors were IrOx potentiometric electrodes especially developed for this work. The acid attack was strong, with the dissolution of the cement/binder phase and formation of gypsum as a product of the reaction. The cement was such an efficient buffer that the pH variation in the mortar was limited to just a small layer in the order of 1–2 mm at the boundary in contact with the acid solution. The corrosion of the iron wires started only when the acidic solution was close to them. The electrochemical results showed the rapid transition between the passive state of iron and its active state with high corrosion rate.

Considering the mechanism of acid attack of reinforced concrete revisited above, it is the concrete that needs protection, among other possible measures, making it compact enough or applying an acid resistant hydrophobic barrier coating.

## Figures and Tables

**Table 1:** Mortar composition.

Mortar Composition	Mass/%
Cement CEM I 42.5N	20.83
Sand 0–2 mm	62.50
Water	16.67
Water/cement ratio	0.8
Sand/cement ratio	3

**Table 2:** Parameters obtained from the impedance spectra in Figure 9.

Depth	R_HF_ (Ω cm^2^)	Y_LF_ (10^−5^ Ω^−1^ s^n^ cm^−2^)	n_LF_	R_LF_ (10^6^ Ω cm^2^)	10^4^ χ^2^
5 mm	1315	4.16	0.840	11.6	19
10 mm	731	4.92	0.829	0.907	9
20 mm	860.7	5.66	0.886	3.18	14
30 mm	1061	5.21	0.850	1.01	29
40 mm	865.7	6.62	0.891	7.31	11

**Table 3:** Parameters obtained from the impedance spectra of Fe wire at 10 mm depth (Figure 10).

Time	R_HF_ (Ω cm^2^)	Y_LF_ (10^−5^ Ω^−1^ s^n^ cm^−2^)	n_LF_	R_LF_ (Ω cm^2^)	10^4^ χ^2^
0 d	2451	4.11	0.810	1.50 × 10^6^	36
25 d	731	4.92	0.829	9.07 × 10^5^	9
26 d (625 h)	698	7.92	0.736	2952	17
27 d (646 h)	425	7.33	0.737	2497	8
27 d (654 h)	156	23.3	0.841	231	1
28 d (678 h)	10.84	48.8	0.829	21.5	3

## Data Availability

The data presented in this study are available on request from the corresponding author.
